# Overexpression of GATA4 enhances the antiapoptotic effect of exosomes secreted from cardiac colony-forming unit fibroblasts via miRNA221-mediated targeting of the PTEN/PI3K/AKT signaling pathway

**DOI:** 10.1186/s13287-020-01759-8

**Published:** 2020-06-26

**Authors:** Chunshu Hao, Zhengri Lu, Yuanyuan Zhao, Zhong Chen, Chengxing Shen, Genshan Ma, Lijuan Chen

**Affiliations:** 1 Department of Cardiology, Zhongda Hospital Affiliated to Southeast University, No. 87 Dingjiaqiao, Gulou District, Nanjing, 210009 China; 2 Medical School of Southeast University, Nanjing, China; 3 Department of Cardiology, Shanghai Jiao Tong University Affiliated Sixth People’s Hospital, Shanghai, China

**Keywords:** GATA4, Exosome, MicroRNA221, PTEN, Apoptosis

## Abstract

**Background:**

GATA4 is an early cardiac-specific transcription factor, and endogenous GATA4-positive cells play a critical role in cardioprotection after myocardial injury. As functional paracrine units of therapeutic cells, exosomes can partially reproduce the reparative properties of their parental cells. Here, we investigated the cardioprotective capabilities of exosomes derived from cardiac colony-forming unit fibroblasts (cCFU-Fs) overexpressing GATA4 (cCFU-Fs^GATA4^) and the underlying mechanism through which these exosomes use microRNA (miRNA) delivery to regulate target proteins in myocardial infarction (MI).

**Methods:**

Exosomes were harvested from cCFU-Fs by ultracentrifugation. miRNA arrays were performed to determine differential miRNA expression between exosomes derived from cCFU-Fs^GATA4^ (GATA4-Exo) and control cCFU-Fs (NC-Exo). A dual-luciferase reporter assay confirmed that miR221 directly targets the 3′ untranslated region (UTR) of the phosphatase and tensin homolog on chromosome ten (PTEN) gene. Cardiac function and myocardial infarct size were evaluated by echocardiography and Masson trichrome staining, respectively.

**Results:**

Compared with NC-Exo, GATA4-Exo increased the survival and reduced the apoptosis of H9c2 cells. Direct intramyocardial transplantation of GATA4-Exo at the border of the ischemic region following ligation of the left anterior descending (LAD) coronary artery significantly restored cardiac contractile function and reduced infarct size. Microarray analysis revealed significantly increased miR221 expression in GATA4-Exo. qPCR confirmed higher miR221 levels in H9c2 cells treated with GATA4-Exo than in those treated with NC-Exo. miR221 mimic-transfected H9c2 cells demonstrated a significantly higher survival rate following exposure to hypoxic conditions than those transfected with miR221 inhibitor. A dual-luciferase reporter gene assay confirmed the PTEN gene as a target of miR221. Western blot analysis showed that H9c2 cells treated with GATA4-Exo exhibited lower PTEN protein expression and higher p-Akt expression.

**Conclusion:**

GATA4 overexpression enhances the protective effect of cCFU-F-derived exosomes on myocardial ischemic injury. In terms of the mechanism, it is at least partly due to the miR221 transferred by GATA4-Exo, which inhibits PTEN expression, activates the phosphatidylinositol 3 kinase (PI3K)/AKT signaling pathway, and subsequently alleviates apoptosis of myocardial cells (CMs).

## Introduction

An important characteristic of acute myocardial infarction (MI) is the large loss of cardiomyocytes (CMs). Cell therapy plays an important role in the MI therapy strategy [1]. However, hypoxic and inflammatory microenvironments make the survival of transplanted cells more difficult [1]. Several studies have demonstrated that cell therapy can improve cardiac function and left ventricle remodeling through paracrine factors. Exosomes (30–150 nm in size) are important paracrine components of many cell types and play an essential role in the shuttling of mRNAs, microRNAs (miRNAs), and proteins between cells [2, 3]. Our previous studies have suggested that exosomes from induced pluripotent stem cells deliver cardioprotective miRNAs and prevent CM apoptosis in the ischemic myocardium [4].

Cardiac colony-forming unit fibroblasts (cCFU-Fs) [5] are a cardiac mesenchymal stem cell (MSC)-like population from the adult murine heart. cCFU-F is a unique heart-derived endothelial cell line that expresses platelet-derived growth factor receptor α (PDGFRα) and stem cell antigen 1 (Sca-1), with multipotent and capable of long-term in vitro growth [6, 7]. Researches over the past several years have suggested that these epicardium-derived cells participate in cardiac development and homeostasis and can migrate into injured areas of the myocardium, where they differentiate into endothelial-like cells after acute ischemic injury [6, 8]. Studies also demonstrated that cCFU-Fs possess repairing functions, including promoting angiogenesis, improving cardiac function after MI, and coordinating inflammatory responses in the aging heart [6, 9, 10]. A recent study confirmed that cCFU-Fs belong to the Tie2 endothelial lineage, with minimal cardiomyogenic potential under both physiological and pathological conditions [11]. It has been proposed that MSCs mediate their properties via the secretion of exosomes containing multiple chemokines, and the delivery of such exosomes can reduce infarct size in animal models [12]. Therefore, it will be interesting to investigate whether and how the paracrine effects contribute to heart repair given the endothelial identity of cCFU-Fs.

During development, the expression of important transcription factors in cCFU-Fs gradually deficient, such as GATA4, which is involved in progenitor cell proliferation, organ morphogenesis during embryogenesis and an essential survival factor in postnatal CMs [13, 14]. Therefore, we believe that the transduction of GATA4 to cCFU-Fs will enhance cell survival and increase tolerance to ischemic environments. Maliken et al. have confirmed that GATA4 is important in endothelial cell maturation and differentiation and suggested that GATA4 could be used as a therapeutic leverage point in affecting endothelial cell biology [15]. Increasing evidence indicates that GATA4 is also one of the antiapoptotic factors regulating cardiac myocyte survival [16–18]. Studies have proved that overexpression of GATA4 not only promotes MSC differentiation but also increases the viability of MSCs in ischemic environments, increases CM survival, and promotes angiogenesis in the ischemic myocardium [19, 20]. However, there are few reports on the potential involvement of GATA4 in the regulation of cell paracrine function. Therapies employing both exosomes and genes hold promise for the treatment of ischemic cardiovascular disease. In particular, cCFU-Fs are excellent carriers of exosome and therapeutic genes to the heart. We hypothesized that GATA-4 overexpression increases cCFU-F paracrine effects that inhibits ischemic induced myocardial injury.

### Ethics statement

All animal protocols carried out in this study were approved by the Institutional Animal Care and Use Committee of Southeast University, and the procedures were conducted in compliance with the National Institutes of Health Guidelines (approval ID: SYXK-2011.3923).

## Methods

### Animals

Male C57BL/6 mice were purchased from the Yangzhou Laboratory Animal Center (Yangzhou, China). The animals were housed under specific pathogen-free conditions under a 12-h light/dark cycle and given free access to food and water. The animal experiment was approved by the Ethics Committee of Southeast University. All efforts were made to minimize animal suffering.

### Cell culture and GATA4 transfection

The H9c2(rat myoblasts) line was obtained from the Zhong Qiao Xin Zhou Biotechnology Co., Ltd. (Shanghai, China) and cultured in 75-cm^2^ flasks in Dulbecco’s modified Eagle’s medium (DMEM) containing 4.5 g/L d-glucose, 1.5 g/L sodium bicarbonate, and 110 mg/L sodium pyruvate, supplemented with 10% fetal bovine serum (Gibco, USA) and penicillin (100 units/ml) and streptomycin (100 μg/ml) in a humidified incubator with 95% air and 5% CO_2_ at 37 °C [21]. The culture medium was changed every 2 or 3 days and then underwent treatments.

cCFU-Fs were isolated from the hearts of 2-month-old male C57BL/6 mice (Yangzhou Laboratory Animal Center) via a previously described procedure [5]. The cCFU-Fs were cultured and maintained in a complete medium consisting of DMEM/F12, 10% fetal calf serum, 200 mM l-glutamine, 55 nM β-mercaptoethanol, and 1% MEM nonessential amino acids. The culture medium was changed every 3 days. GV208 lentivirus (GeneChem, Co., Ltd., Shanghai, China) carrying GATA4 was used to transduce cCFU-Fs using polybrene as the linker molecule. qPCR was utilized to confirm the expression of GATA4. The reaction mixture contained dNTPs (2.5 mM each), forward and reverse primers (10 μM each; Shanghai Genechem Co., Ltd., Shanghai, China), template (10 ng/μL), and PrimeSTAR HS DNA polymerase (0.5 μL). When the transduction efficiency exceeded 80%, the multiplicity of infection (MOI) was measured. The required MOI was 20–50. Transduction with the lentivirus vector using the same protocol was also performed as a control (Table 1).

**Table 1:** Primer sequences

Primer	Sequence	
miR221mimics	Forward	AGCUACAUUGUCUGCUGGGUUUC
	Reverse	AACCCAGCAGACAAUGUAGCUUU
miR221 mimics NC	Forward	UUC UCCGAACGUGUCACGUTT
	Reverse	ACGUGACACGUUCGGAGAATT
miR221 inhibitor	Forward	GAAACCCAGCAGACAAUGUAGCU
miR221 inhibitor NC	Forward	CAGUACUUUUGUGUAGUACAA

### Exosome purification

Exosomes were obtained from the cCFU-F culture supernatants by ultracentrifugation. Briefly, cCFU-Fs were cultured for 48 h in DMEM/F12 with 2% exosome-free FBS (Exo-FBS, System Biosciences (SBI), Mountain View, CA) and then centrifuged to harvest the supernatant. The supernatants were centrifuged for 45 min at 3000×*g*, passed through a 0.22-μm filter to remove debris, and then centrifuged at 200,000×*g* (Beckman Coulter) for 2 h at 4 °C. After discarding the supernatants, the pellets were washed in PBS, resuspended, and centrifuged at 200,000×*g* for 2 h at 4 °C. The purified exosome fraction was resuspended in 50 μl of PBS and stored at − 80 °C.

### Transmission electron microscopy (TEM) observation

After fixation with 4% paraformaldehyde and 1% glutaraldehyde in a 0.1 M sodium cacodylate buffer (pH 7.2) for 3 h at room temperature, the cells were washed with cacodylate buffer, postfixed in 1% osmium tetroxide, dehydrated stepwise in a graded ethanol series (50–100%), and embedded in Epon. Thin (1-mm thick) and ultrathin (70–80-nm thick) sections were cut from the polymer with a Reichert (Depew, NY, USA) Ultracut S microtome, placed on copper grids, and quickly stained with uranyl acetate and lead citrate. The exosomes were fixed with 2% paraformaldehyde, loaded on 300-mesh formvar/carbon-coated electron microscopy grids, postfixed in 1% glutaraldehyde, and then contrasted and embedded. TEM images were obtained with a FEI Tecnai Spirit G2 transmission electron microscope (Philip, Amsterdam, Netherlands, ca #CM-120) operating at 120 kV.

### Nanoparticle tracking analysis (NTA)

A total of 20 μg of exosomes was dissolved in 1 ml of PBS and rotated for 1 min to maintain an even distribution. Subsequently, a nanoparticle tracking analyzer (NanoSight, Amesbury, Wilkshire, UK) was utilized to accurately measure the diameter of the exosomes and further applied to analyze and save the data.

### miRNA chip experiment and analysis

Total RNA was extracted from cCFU-F-Exos by using a Qiagen miRNeasy Mini Kit. Exosome replicas were derived from different cCFU-F isolates. Global miRNA expression patterns were then examined by using Affymetrix GeneChip miRNA 4.0 arrays. The Affymetrix GeneChip miRNA 4.0 array was designed based on miRBase version 20 (www.mirbase.org). One microgram of total RNA was used as the input of the labeling reaction as recommended by the protocol of the Genisphere FlashTag Biotin RNA Labeling Kit. Labeled miRNA was then hybridized to the array at 60 rpm for 16 h at 48 °C. The gene chips were then scanned by using a Hewlett Packard Gene Array Scanner G3000 7G (Affymetrix). Expression data were generated by using Affymetrix Expression Console software and normalized according to the MAS5 method. The random variance model (RVM) *t* test was applied to filter the differentially expressed genes between the control and experimental groups. Differentially expressed miRNAs were defined based on a *p* value threshold and fold-change analysis. The criteria for significantly differentially expressed miRNAs were a *p* value < 0.05 and a fold change in expression of at least 2.0. Unsupervised hierarchical clustering was performed by using Cluster 3.0 and Java TreeView software.

### miRNA target prediction and luciferase reporter assay

The PicTar algorithm (http://pictar.mdc-berlin.de) was used to confirm miRNA-binding sites in mouse phosphatase and tensin homolog on chromosome ten (PTEN). The mouse 3′ untranslated region (UTR) of the PTEN gene was amplified by PCR using the following primers: PTEN-3′ UTR-Forward: 5′-ACCAGGACCAGAGGAAACCT-3′, and PTEN-3′ UTR-Reverse: 5′-TTTGTCAGGGTGAGCACAAGA-3′. The cDNA was cloned into the XbaI/XbaI site of the pGL3-Control vector (Promega, USA) downstream of the luciferase gene to generate the pGL3-PTEN vector. For the luciferase reporter assay, T293 cells (Zhong Qiao Xin Zhou Biotechnology, Shanghai, China) were cultured in 96-well plates and transfected with 0.2 μg of the pGL3-PTEN or pGL3-Control plasmid and 5 pmol of AS-miRNAs (AS-miR-221) using Lipofectamine 2000. At 48 h after transfection, luciferase activity was measured using the Luciferase Assay System (Promega). The primers used are listed in Table 1.

### Quantification of mRNA and miRNA levels

Total RNA was extracted from cCFU-Fs or isolated cCFU-F exosomes by RNAzol RT (Invitrogen) following the manufacturer’s instructions. The RNA concentrations were determined by using a NanoDrop ND-1000 spectrophotometer (Thermo Scientific). Isolated RNAs were polyadenylated using an NCode miRNA First-Strand cDNA Synthesis Kit (Invitrogen). qRT-PCR was performed with SYBR Green I (DH Biotech, Shanghai) and Prism 7500 SDS (Applied Biosystems; Thermo Fisher Scientific, Inc.). Amplification was performed at 95 °C for 10 min, followed by 40 cycles at 95 °C for 15 s and 60 °C for 1 min. For mature miRNA expression, the universal primer provided in the NCodeTM miRNA First-Strand cDNA Synthesis Kit was used with one of the following forward primers:

Mmu-miR221: 5′-GUCAACAUCAGUCUGAUAAGCUA-3′and Mmu-miRU6: 5′-ACACGCAAATTCGTGAAGC-3′.

The relative gene expression values were calculated using the ΔΔCt method (ΔΔCt = ΔΔCt treated- ΔΔCt untreated control) with the equation *y* = 2-ΔΔCt, and U6 was used as a control.

### Myocardial ischemia model and exosome implantation

Acute regional left ventricular (LV) MI was generated by permanent ligation of the left anterior descending (LAD) coronary artery. Briefly, male C57BL/6 mice weighing 25 to 30 g were anesthetized with chloral hydrate (10%, 0.1 ml/20 g) and mechanically ventilated. The chest was opened along the fourth intercostal space on the left, and the heart was exposed through pericardiotomy. The root of the LAD coronary artery was ligated near its origin with a 6–0 Prolene suture. In vivo delivery of exosomes (GATA4-Exo, NC-Exo, GATA4-Exo + miR221 inhibitor), miR221 mimics (GeneChem, Co., Ltd., Shanghai, China) into ischemic hearts with the chest open. The dosages used per mouse were 80 ng miR221 mimics, and exosomes (100 μg in 100 μl of PBS) were injected along the border between infarct zone and normal myocardium after LAD. The control group consisted of sham-operated mice that had undergone all surgical procedures except LAD ligation. Then, the chest was closed. Intraperitoneal injection of penicillin was performed postoperatively.

### Detection of cardiac function and myocardial infarct size

Mice were anesthetized with 1.0% inhaled isoflurane, and echocardiography was performed with a 12-MHz transducer (SONOS 7500; Philips Medical Systems) to evaluate the cardiac function at 28 days after MI; this procedure was repeated 3 times with the same equipment in the same examiner. Under electrocardiograph (ECG) monitoring of heart rate, 2D images of the hearts at the level of the greatest LV diameter were acquired in long-axis views. The LV ejection fraction (LVEF) was measured from 2D long-axis views of the infarcted area. Twenty-eight days after MI, the animals were euthanized to remove the hearts, and the infarct size was determined with Masson trichrome staining (Sigma). In brief, the hearts were simply trimmed to eliminate the upper part of the ligated area after immersion in 4% formaldehyde for 48 h. The remainder of the heart was cut into approximately 5-mm specimens perpendicular to the axis of the LAD coronary artery that were mounted on plastic and processed with paraffin embedding, producing 4-μm paraffin tissue sections. To measure the severity of myocardial fibrosis, the slices were stained with Masson trichrome to measure the average ratio of the fibrotic area to the entire LV cross-sectional area (fibrosis area %) using ImageJ software.

### Terminal deoxynucleotidyl transferase (TdT)-mediated deoxyuridine triphosphate (dUTP)-biotin nick end-labeling (TUNEL) staining

To quantify apoptotic CMs, mouse hearts were removed 24 h after MI. Apoptosis in the myocardial tissue was assessed using the DeadEnd™ Fluorometric TUNEL System (Promega) per the manufacturer’s protocol. Images were taken on a Nikon Eclipse T*i* confocal microscope (Nikon 246, Tokyo, Japan) with a 20× objective. Five visual fields were randomly selected from each section, and the average number of apoptotic cells/200 cells was assessed. The apoptotic index (AI) was determined by the formula AI = (number of positive cells/total number of counted cells) × 100%.

### Statistical analysis

All data were analyzed using SPSS 21.0 software and tested by analysis of the normality of distribution and application of the homogeneity test of variance. Measurement data are expressed as the mean ± standard deviation (SD). An independent sample *t* test was used for comparisons between two groups. Comparisons of data among multiple groups obeying a normal distribution were carried out by one-way analysis of variance (ANOVA). Differences for which *p* < 0.05 were considered statistically significant.

## Results

### Characterization of cCFU-Fs^GATA4^ and cCFU-F exosomes

**Fig. 1:**
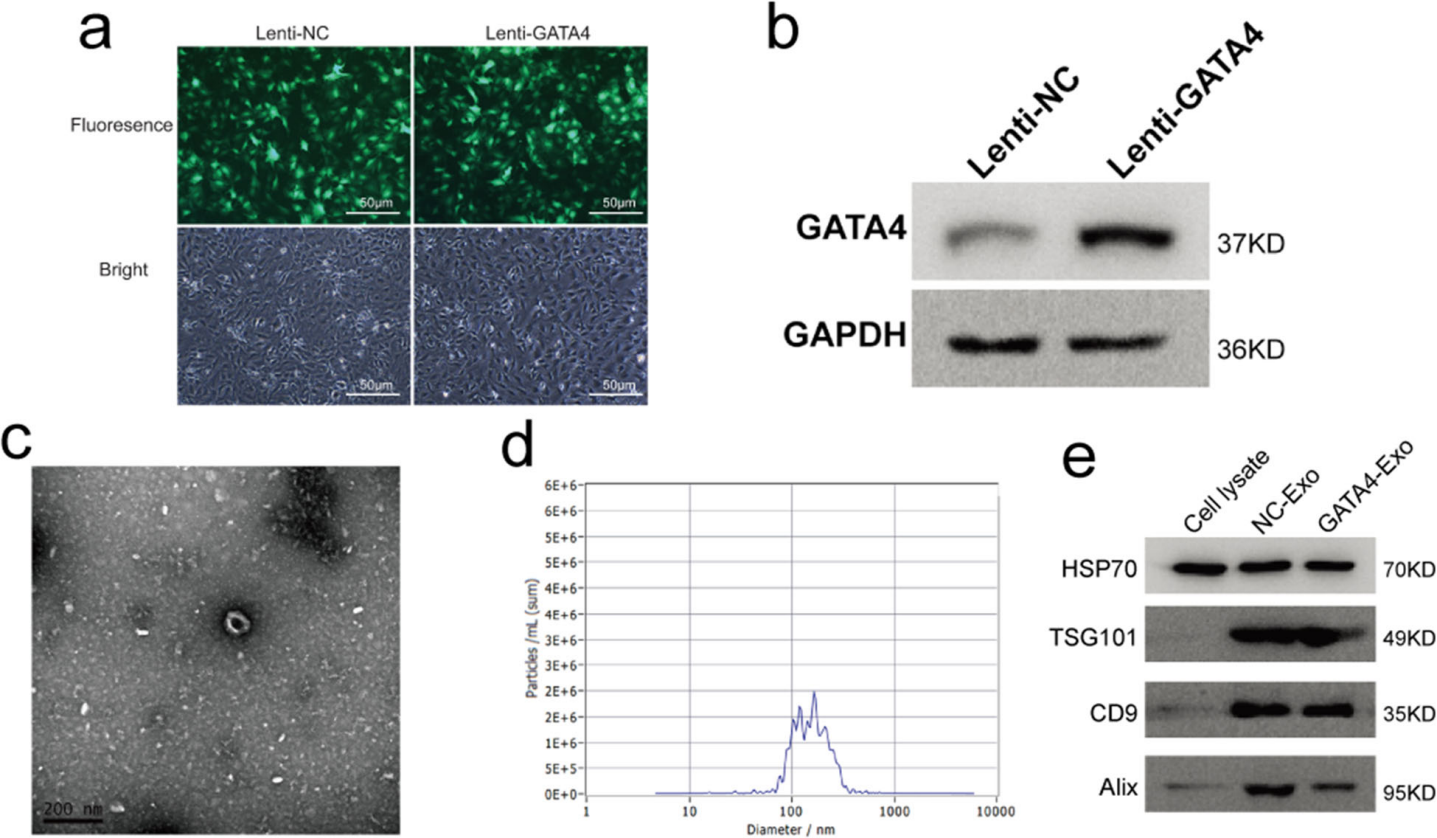
Characterization of cCFU-Fs transduced with GATA4 (cCFU-Fs^GATA4^) and identification of exosomes from cCFU-Fs. **a** Immunostaining of cCFU-Fs transduced with GATA4 (cCFU-Fs^GATA4^) or empty vector (cCFU-Fs^NC^, control). GFP was expressed in both cCFU-Fs^NC^ and cCFU-Fs^GATA4^. Scale bar: 50 μm. **b** Western blot of GATA4 in cCFU-Fs^GATA4^ and cCFU-Fs^NC^ (*n* = 3). **c** TEM image of exosomes (*n* = 6). Scale bar: 200 nm. **d** NanoSight analysis of the particle size (*n* = 4). **e** Detection of CD9, TSG101, and Alix by Western blotting (*n* = 3)

Lentivirus-mediated transduction and expression of the GFP-GATA4 bicistronic construct were verified by immunostaining and Western blotting. Both cCFU-Fs^GATA4^ and cCFU-Fs transfected with the empty vector control (cCFU-Fs^NC^) expressed GFP, and there was no difference in cellular morphology between cCFU-Fs^GATA4^ and cCFU-Fs^NC^ (Fig. 1a). Western blot data indicated that the expression of GATA4 was much higher in cCFU-Fs^GATA4^ (Fig. 1b). The morphology of the cCFU-F-derived exosomes was directly acquired under a transmission electron microscope, and the particles were observed to be small, round vesicles with a bilayer membrane structure, and diameter of approximately 110 nm (Fig. 1c). NanoSight analysis demonstrated that the diameters of the particles were within the range of 50–200 nm, with an average of 145 nm (Fig. 1d). We measured the protein levels of the exosome markers TSG101, Alix, and CD9 by Western blotting, and all markers could be detected in the cCFU-F-derived exosomes (Fig. 1e). Thus, the above results indicated that the cCFU-F-derived particles obtained in our experiments were exosomes.

### cCFU-F^GATA4^-derived exosomes (GATA4-Exo) reduced the apoptosis of H9c2 cells induced by hypoxia

**Fig. 2:**
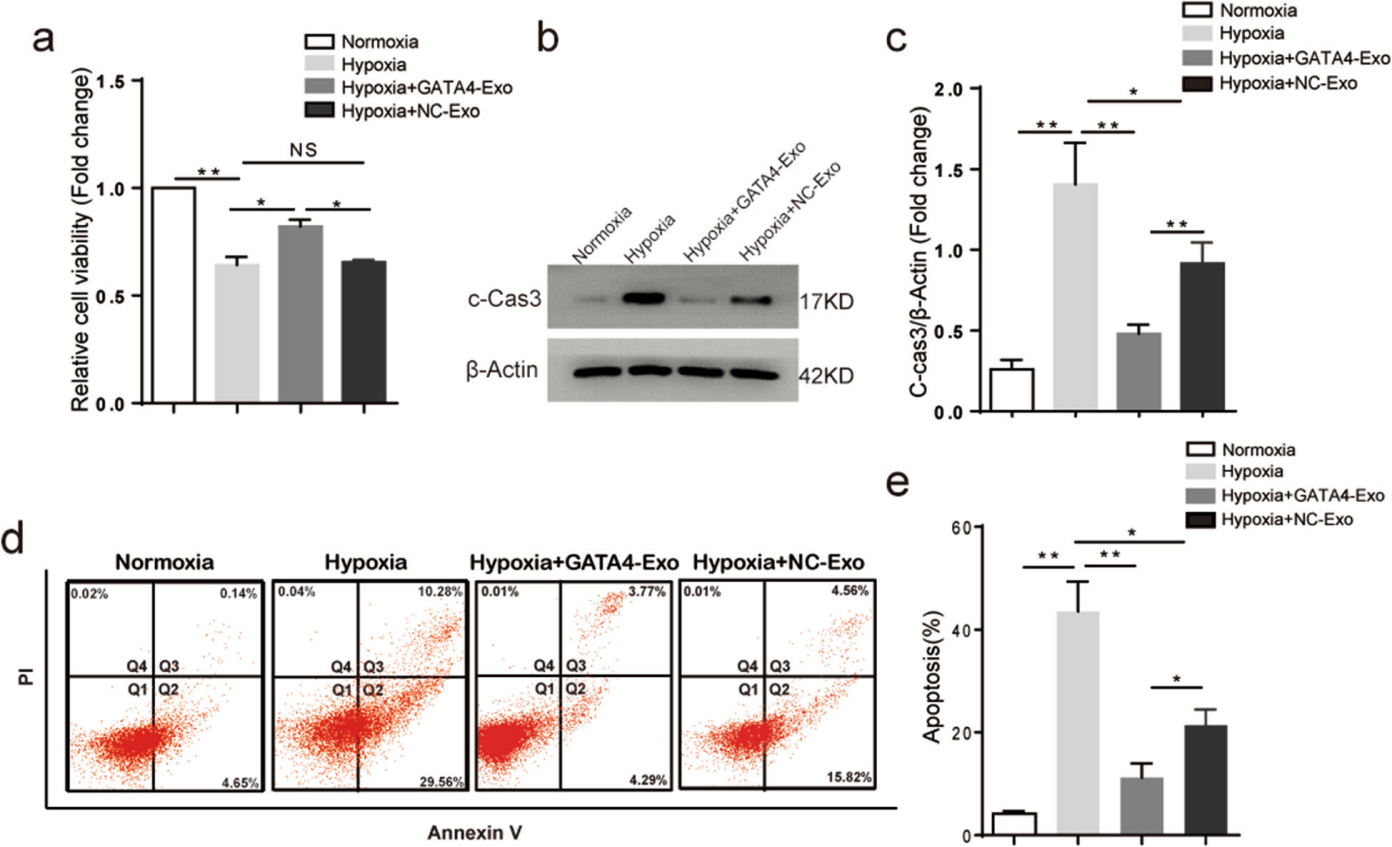
The effect of cCFU-Fs^GATA4^ exosomes on hypoxia-induced cardiomyocyte injury. **a** Cell viability of H9c2 cells after coculture with GATA4-Exo or NC-Exo under hypoxic conditions for 24 h was analyzed by CCK8 assay (*n* = 4, **p < 0.01 for hypoxia vs normoxia; *p < 0.05 between the indicated groups). **b**, **c** Western blot analysis of c-caspase-3 in H9c2 cells after exposure to hypoxia, GATA4-Exo, or NC-Exo (*n* = 3, **p < 0.01 for hypoxia vs normoxia and hypoxia vs GATA4-Exo; *p < 0.05 for GATA4-Exo vs NC-Exo). **d**, **e** Apoptotic H9c2 cells in response to hypoxia and coculture with GATA4-Exo or NC-Exo are determined by flow cytometry (Q1:viable cells, Q2: early apoptotic cells, Q3: late apoptotic cells, Q4: Necrotic cells, *n* = 3) (c-Cas-3: cleaved-caspase-3). (*n* = 3, *p < 0.05, **p < 0.01 using two-tailed Student’s *t* test. Data are represented as mean and SEM)

To compare the protective effect of the exosomes from cCFU-Fs^GATA4^ and cCFU-Fs^NC^ on hypoxia-induced myocardial cell injury, we measured the cell viability of H9c2 cells after coculture with the two kinds of exosomes under hypoxic conditions for 24 h using a CCK8 kit. The results showed that the cell viability of the H9c2 cells was suppressed after hypoxia treatment. The addition of exosomes (100 μg/ml) generated from cultured cCFU-Fs^NC^ has no pronounced protective effect on cell damage caused by hypoxia, but the GATA4-Exo can significantly increase cell viability. (Fig. 2a, *n* = 3). Compared with NC-Exo pretreatment, GATA4-Exo treatment significantly decreased the protein expression of cleaved-caspase-3 (c-Cas-3) in hypoxia-stimulated H9c2 cells, indicating that GATA4-Exo attenuated hypoxia-induced apoptosis (Fig. 2b, c; *n* = 3). Similarly, the flow cytometry results showed that both NC-Exo and GATA4-Exo pretreatment can reduce the percent of early (Q2) and late (Q3) apoptotic cells caused by hypoxia. Compared with NC-Exo, the reduction caused by GATA4-Exo was more remarkable, especially for the percentage of early apoptotic cells (Fig. 2d, e; *n* = 3). These results indicate that GATA4 is involved in the protective effects of GATA4-Exo against hypoxia-induced myocardial cell injury.

### Intramyocardial delivery of GATA4-Exo preserves cardiac function and reduces infarct size by inhibiting cardiomyocyte apoptosis

**Fig. 3:**
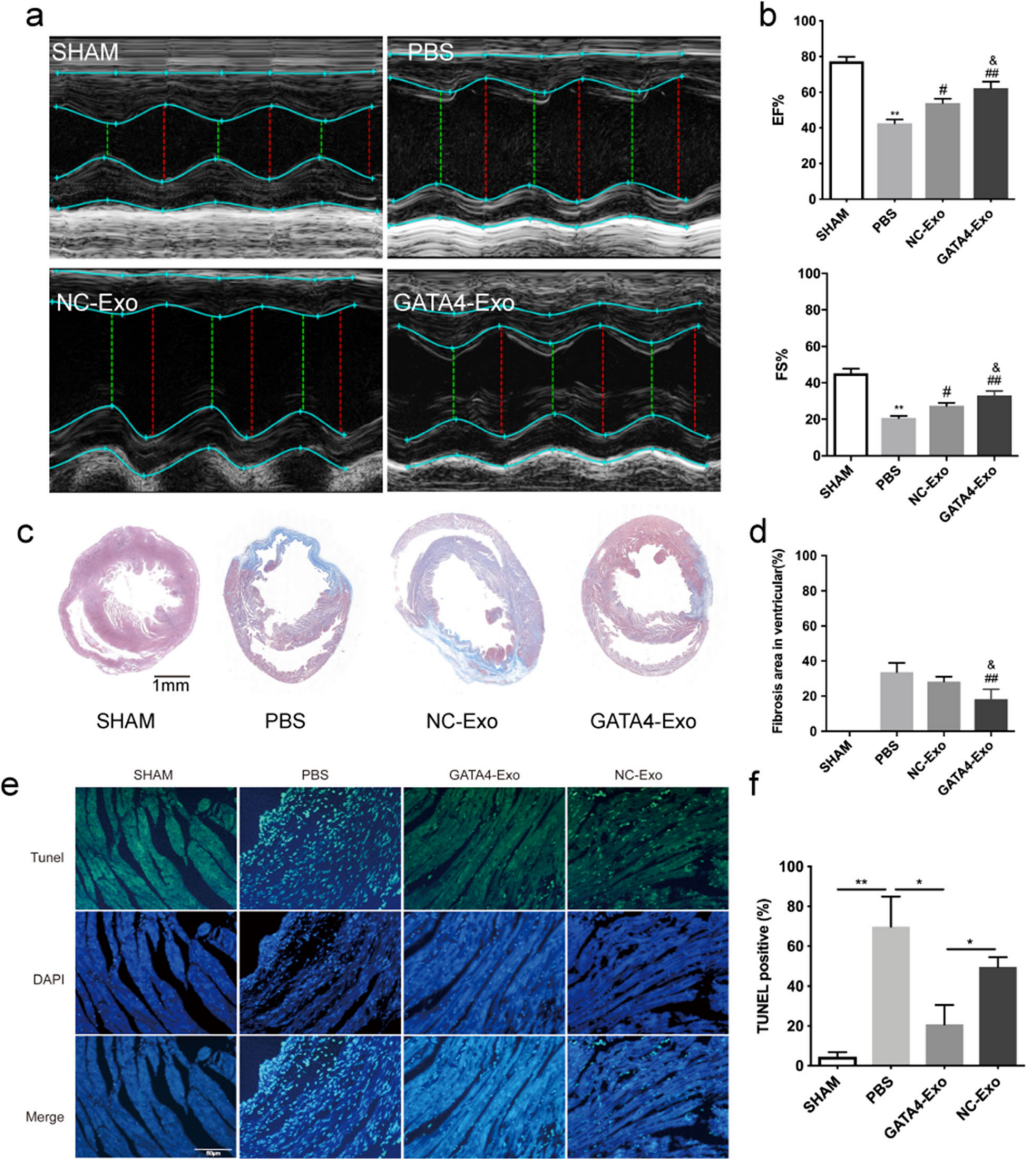
Enhanced cardiac function and decreased apoptotic cells after myocardial infarction in mice transplanted with GATA4-Exo. **a**, **b** Representative M-mode images (**a**) and quantification (**b**) of EF% and FS% measured by echocardiography of sham (*n* = 6), PBS-treated (*n* = 6), NC-Exo-treated (*n* = 6), and GATA4-Exo-treated (*n* = 6) mice at 28 days after MI. (**p < 0.01 for PBS vs SHAM, #*p* < 0.05 for NC-Exo vs PBS, ##*p* < 0.01 for GATA4-Exo vs PBS, & *p* < 0.05 for GATA4-Exo vs NC-Exo). **c**, **d** Masson trichome-stained myocardial sections at 28 days after MI in mice treated with PBS, NC-Exo, or GATA4-Exo (*n* = 6 mice per experimental group, for each sample, 4 to 6 slices were taken according to the size of the heart). Scar tissue and viable myocardium are identified in blue and red, respectively. The percentage of the fibrotic area after AMI was calculated using the software ImageJ. The bar graph on the right quantifies the extent of fibrosis in each treatment group (##*p* < 0.01 for GATA4-Exo vs PBS, & *p* < 0.05 for GATA4-Exo vs NC-Exo). Scale bar: 1 mm. EF%: ejection fraction %; FS%: fraction shortening %. **e** Immunohistochemistry of sham-, PBS-, GATA4-Exo-, or NC-Exo-treated heart sections marking TUNEL-positive cardiomyocytes within the border zone of the infarcted hearts at 24 h after LAD artery ligation. Scale bar: 50 μm. **f** Quantification of myocardial apoptosis. Green staining indicates TUNEL-positive cells (*p < 0.05, **p < 0.01 between the indicated groups, *n* = 6)

To evaluate the therapeutic effect of GATA4-Exo, we generated a mouse model of acute MI via permanent LAD artery ligation. We intramyocardially injected 100 μl of PBS, NC-Exo, or GATA4-Exo into the mice after LAD ligation, and mice in the sham operation groups served as controls. Echocardiography showed that GATA4-Exo treatment preserved LV function, as indicated by the end-systolic chamber volume (ESCV) and end-systolic left ventricular internal diameter (LVID) after myocardial infarction, but NC-Exo treatment did not. Similarly, GATA4-Exo treatment improved systolic function, as indicated by the higher ejection fraction (EF%) and fraction shortening (FS%) (Fig. 3a, b) (*p* < 0.01 for PBS vs. sham; *p* < 0.05 for NC-Exo vs. PBS, *p* < 0.01 for GATA4-Exo vs. PBS; *p* < 0.05 for GATA4-Exo vs. NC-Exo). To evaluate the size of the MI, Masson trichrome staining was performed 28 days after exosome transplantation. In Fig. 3c, red indicates normal myocardium, while blue indicates fibrotic tissue. Through quantitative analyses using ImageJ software, we found that the percentage of the fibrotic area in the entire LV cross-sectional area was distinctly reduced in the GATA4-Exo group compared with the NC-Exo and PBS groups (Fig. 3c, d) (*p* < 0.01 for GATA4-Exo vs. PBS; *p* < 0.05 for GATA4-Exo vs. NC-Exo).

Acute ischemia leads to apoptotic signal activation in CMs. TUNEL staining was undertaken to determine whether GATA4-Exo treatment could better protect against acute the MI-induced apoptosis of CMs in vivo. The results showed that the number of apoptotic cells in the border zone of the ischemic heart tissue was significantly increased in PBS-injected mice compared with mice in the sham operation group (Fig. 3e, f). Delivery of GATA4-Exo to the myocardium after LAD artery ligation caused a 49% reduction in TUNEL-positive apoptotic cells compared with those in the hearts of PBS-treated control mice (*p* < 0.05), while NC-Exo delivery caused a 20.25% reduction (*p* = 0.0625). These data illustrate that functional improvement in the GATA4-Exo group was accompanied by decreased infarct size which was partly due to the inhibition of CM apoptosis.

### miRNA221 carried by GATA4-Exo is critical for GATA4-Exo-mediated protection of injured H9c2 cells

**Fig. 4:**
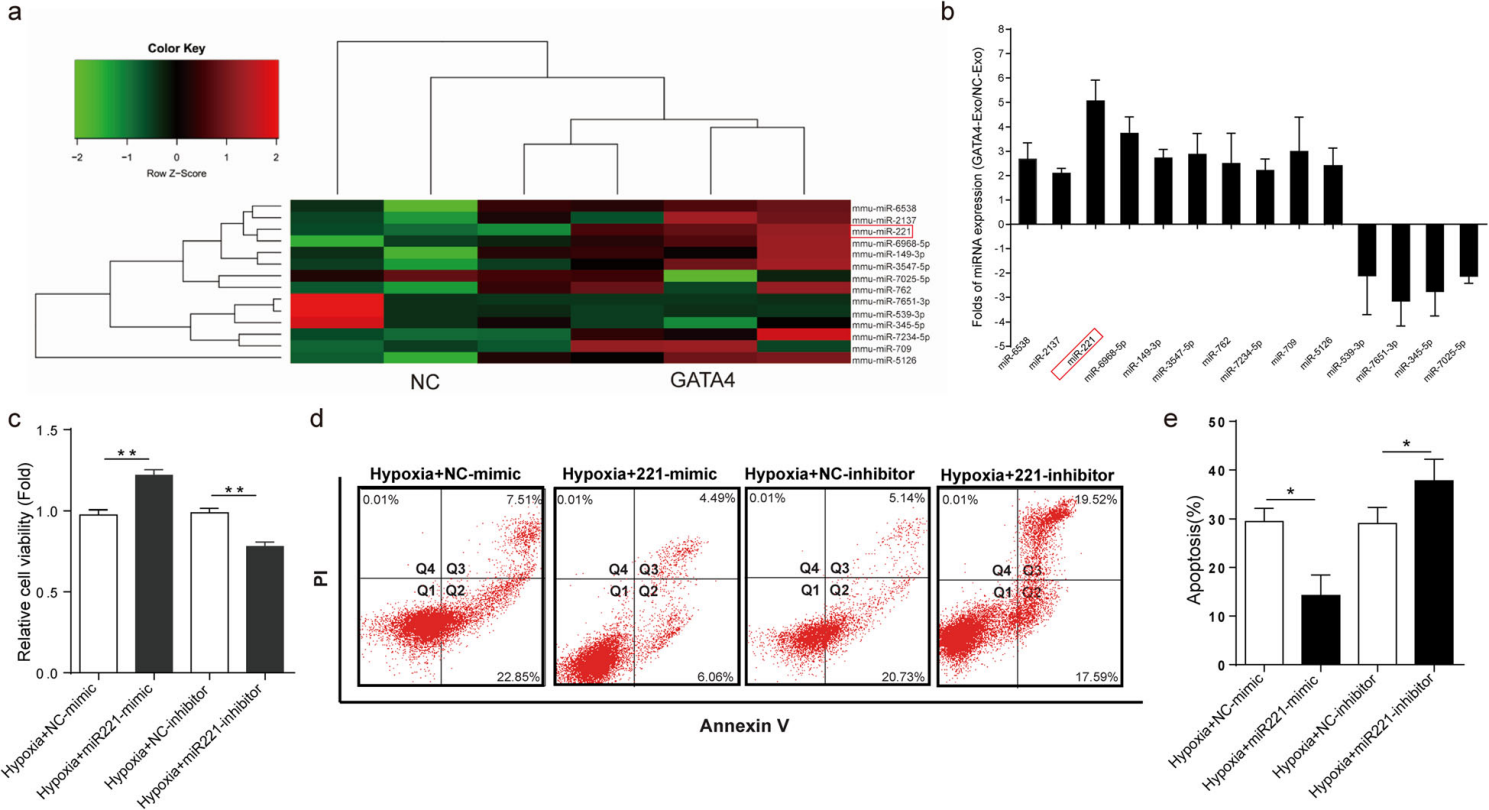
MicroRNA (miRNA) expression profiling and the effect of miR221 on hypoxia-induced cardiomyocyte injury. **a** Heat map and supervised hierarchical clustering of the 10 upregulated and 4 downregulated miRNAs with *a* ≥ 2-fold difference in expression between GATA4-Exo and NC-Exo (triplicate assays were done for each exosomes replica from different cCFU-F isolates). **b** Validation of microarray data using real-time PCR, and the relative expression of miRNAs in GATA4-Exo compared to NC-Exo (*n* = 3). **c** The cell viability of H9c2 cells after coculture with miR221 mimics or miR221 inhibitor under hypoxic conditions for 24 h was analyzed by CCK8 assay (*n* = 4, **p < 0.01 for comparisons between groups). **d**, **e** Apoptotic H9c2 cells in response to hypoxia and coculture with a miR221 mimics or miR221 inhibitor determined by flow cytometry (*n* = 3, *p < 0.05 for comparisons between groups)

We used an unbiased approach to further investigate whether GATA4 overexpression affects miRNA species in cCFU-F-derived exosomes by performing exosomal miRNA array analysis. The hierarchical clustering of miRNA expression (Fig. 4a) showed that GATA4-Exo had a miRNA expression signature very different from that of NC-Exo (*n* = 3). Overall, 10 miRNAs, including miR6538, miR2137, miR221, and miR6968-5p, showed marked upregulation in GATA4-Exo compared with NC-Exo, whereas 4 miRNAs (miR7651-3p, miR539-3p, miR345-5p, miR7025-5p) showed evident downregulation in GATA4-Exo compared with NC-Exo. Differential miRNA expression in the networks was further validated by real-time quantitative polymerase chain reaction. All tested miRNAs exhibited an agreement with the array-generated expression data. Compared with other miRNAs, miR-221 has the most significantly increase in GATA4-Exo (Fig. 4b). Therefore, we focused on miRNA221, which plays important roles in cell proliferation, apoptosis, and differentiation [22, 23]. To investigate the role of miR221 in H9c2 cells in response to hypoxia, a miR221 mimics and a miR221 inhibitor were constructed. The results suggested that the miR221 mimics could protect cells against hypoxia-induced H9c2 cell injury, while the miR221 inhibitor aggravated the damage (Fig. 4c, *n* = 4). The treatment of H9c2 cells with the miR221 mimics decreased hypoxia-induced apoptosis (Fig. 4d, *n* = 3). However, the inhibitor increased cell injury, in agreement with the flow cytometry results. Annexin V analysis showed significantly more apoptotic cells among cells transfected with the miR221 inhibitor than among those transfected with the miR221 mimics (Fig. 4d, e). Furthermore, in vivo study, we confirmed that GATA4-Exo + miR221inhibitor not only weakened the role of GATA4-Exo in improving cardiac function and reducing fibrosis, but also neutralized the anti-apoptotic effect GATA4-Exo (Fig. S3). These data demonstrated that the cardioprotective effect of GATA4-Exo might be comodulated by miR221.

### miRNA221 inhibits the apoptosis of H9c2 cells by targeting PTEN

**Fig. 5:**
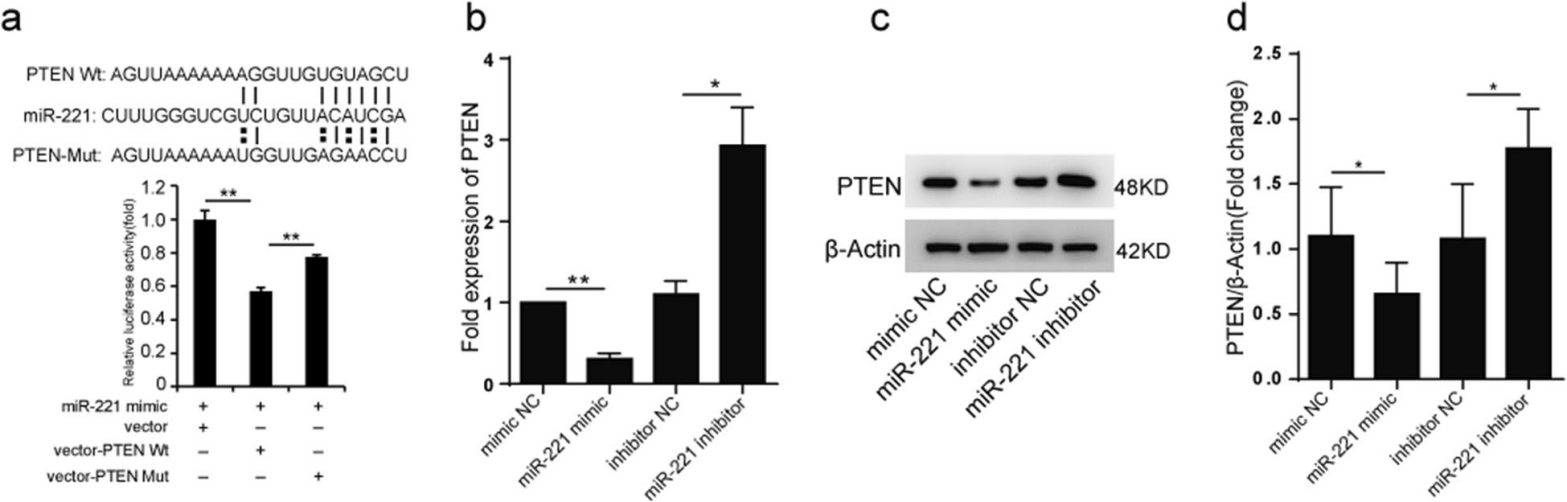
PTEN is a target gene of miR221. **a** Luciferase reporter assays were performed using the wild-type PTEN 3′ UTR sequence containing either the seed sequence for miR221 recognition (PTEN-3′ UTRwt) or a mutated 3′ UTR (PTEN-3′ UTRmut). Luciferase activity was determined 48 h after transfection. The ratio of normalized luciferase activities is shown (*n* = 3). **b** Real-time PCR analysis of PTEN expression in H9c2 cells after treatment with a miR221 mimics or inhibitor (*n* = 3). **c**, **d** The protein levels of PTEN in H9c2 cells as determined by Western blotting (*n* = 3, **p < 0.01, *p < 0.05 for comparisons between groups)

Having established that GATA4-Exo can confer cardioprotection through the upregulation of miR221, we sought to further determine the mechanism by which miR221 protects the cells. By using http://www.targetscan.org/, PUMA, PTEN, DKK, Bim, Foxo3, and other genes were predicted to be the target genes of miR221. We pay attention to the tumor suppressors PTEN which also correlated with cell apoptosis [24]. PTEN downregulates cell proliferation and promotes apoptosis by suppressing Akt activation [25]. To examine whether miRNA221 contains sites that directly bind PTEN mRNA, luciferase reporter assays were performed. Briefly, wild-type PTEN 3′ UTR sequences containing either the seed sequence for miR221 recognition (PTEN-3′ UTRwt) or a mutated 3′ UTR (PTEN-3′ UTRmut) were constructed. Compared with the NC mimics, the relative luciferase activity of the PTEN-3′ UTRwt reporter was significantly reduced by the miR221 mimics; however, mutation of the miR221 seed sequence reversed this inhibitory effect (Fig. 5a, *n* = 3). Therefore, the 3′ UTR of PTEN contains the direct binding seed of miR221. Additionally, the miR221 mimics downregulated the expression of PTEN at both the mRNA and protein levels, and conversely, the inhibitor upregulated the expression of PTEN (Fig. 5b–d). Together, these data demonstrate that PTEN is a target gene of miR221 (*n* = 3).

### The PTEN-PI3K/Akt signaling pathway is involved in the antiapoptotic effect of GATA4-Exo

**Fig. 6:**
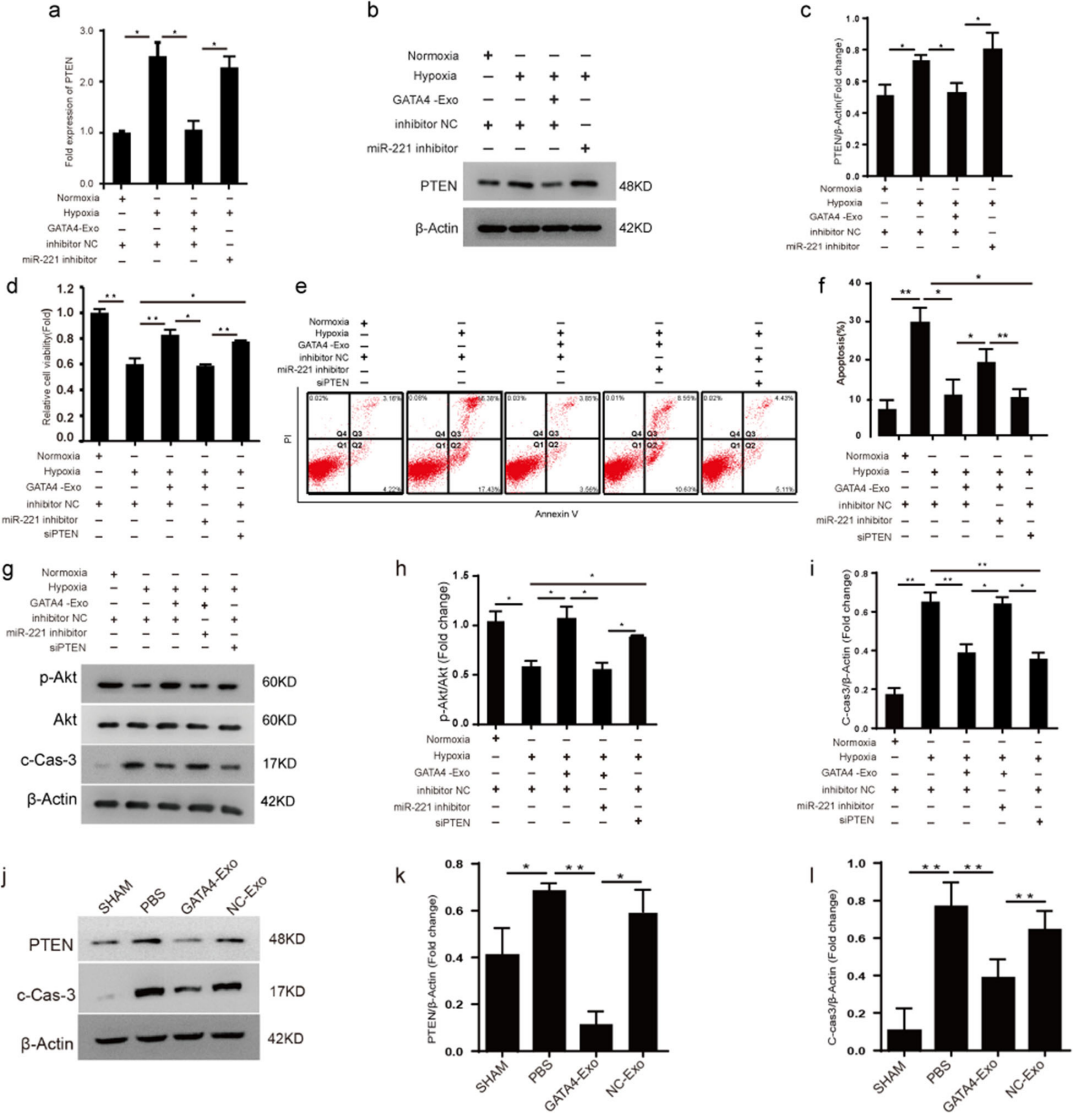
The PTEN-PI3K/Akt signaling pathway is involved in the antiapoptotic effect of GATA4-Exo. **a**–**c** PTEN mRNA and protein levels after treatment with hypoxia, GATA4-Exo, or miR221 inhibitor were determined by qPCR and Western blotting, respectively (*n* = 3, **p < 0.01 for comparisons between groups). **d** The cell viability of H9c2 cells after treatment with hypoxia, GATA4-Exo, miR221 inhibitor, and/or siPTEN was analyzed by CCK8 assay (*n* = 3, **p < 0.01 for comparisons between groups). **e**, **f** Apoptotic H9c2 cells in response to hypoxia, GATA4-Exo, miR221 inhibitor, and siPTEN were determined by flow cytometry (*n* = 3, *p < 0.05, **p < 0.01 for comparisons between groups). **g**–**i** Western blot analysis of p-Akt, Akt, and c-caspase-3 in H9c2 cells after exposure to hypoxia, GATA4-Exo, miR221 inhibitor, and/or siPTEN. Relative expression was determined following normalization to β-actin levels (*n* = 3, *p < 0.05, **p < 0.01 for comparisons between groups). All data were obtained from 3 independent experiments. **j**–**l** The protein expression levels of PTEN and c-caspase-3 within the myocardium. Relative expression was determined following normalization to β-actin levels (*p < 0.05, **p < 0.01 between indicated groups, *n* = 6)

Previous studies have reported that the PTEN-phosphatidylinositol 3-kinase (PI3K)/Akt pathway plays an important role in the process of apoptosis [26, 27]. We hypothesized that PTEN is also involved in the cardioprotective effect of GATA4-Exo. The results showed that GATA4-Exo significantly downregulated the expression of PTEN at both the mRNA and protein levels, which was in contrast with the effect of the miR221 inhibitor (Fig. 6a–c). In addition, to examine the role of PTEN in the antiapoptotic effect of GATA4-Exo in H9c2 cells, we knocked down PTEN with siRNA. The results of CCK8 assays showed that GATA4-Exo protected H9c2 cells from apoptosis and injury caused by hypoxia, while the miR221 inhibitor decreased this protective effect (Fig. 6d–f). When PTEN was knocked out with siRNA in H9c2 cells, the cells showed reduced apoptosis and increased cell viability after hypoxia stimulation, which is similar to the effects of GATA4-Exo (Fig. 6d–f). Furthermore, molecular signaling associated with PTEN was assessed. The PI3K/Akt pathway, the main cellular pathway that promotes survival and growth, is a critical pathway downstream of PTEN [26, 28, 29]. We tested the effects of GATA4-Exo, the miR221 inhibitor, and PTEN deletion on p-Akt and c-Cas-3 expression after hypoxia stimulation (Fig. 6g–i). The results suggested that hypoxia downregulated the expression of p-Akt but increased c-Cas-3 expression. The expression of p-Akt was increased, while the expression of c-Cas-3 was reduced in cells incubated with GATA4-Exo before hypoxia compared with cells treated with hypoxia alone. The pretreatment of the miRNA221 inhibitor abrogated the effect of GATA4-Exo. When cells were pretreated with siPTEN, the changes in p-Akt and c-Cas-3 expression were similar to those in the GATA4-Exo-cotreated group. These outcomes suggest that PTEN promotes H9c2 cell apoptosis induced by hypoxia through the PI3K/Akt-caspase-3 signaling pathway. Similarly, in vivo studies, compared with NC-Exo injection, GATA4-Exo significantly downregulated the protein expression of PTEN and c-Cas-3 in the acute MI model (Fig. 6j–l, *n* = 6). Furthermore, in order to demonstrate the role of miR221 after GATA4-Exo transplantation in vivo, we evaluated the level of miR221 after administration of two exosomes in left ventricular tissue by real-time PCR. As a result, the increased expression of miRNA221 was observed in both NC-Exo and GATA4-Exo administration, and the degree of GATA4-Exo was more significant (Fig. S1), which indicates that the restoration of heart function of exosome derived from c-CFU-Fs^GATA4^ is achieved partly by miR221. These results indicated that the protective effects of GATA4-Exo against LAD ligation-induced myocardial cell apoptosis were mediated by GATA4.

## Discussion

This study reveals that GATA4-mediated regulation of miR221 in cCFU-F exosomes is associated with the cardioprotective effects of the exosomes. Three key conclusions are reported in the present study: (1) GATA4-Exo increased the tolerance of H9c2 cells to hypoxic injury, promoted cardiac functional recovery, and reduced infarct size. (2) GATA4 upregulated the expression of many miRNAs in exosome-derived cCFU-Fs that are related to antiapoptotic effects, specifically miR221. (3) miR221 transferred by GATA4-Exo downregulated the expression of target proteins, especially PTEN, and activated the PI3K/Akt signaling pathway.

Cell transplantation has been widely used in the treatment of myocardial infarction in previous decades. cCFU-Fs are unique heart-derived endothelial lineage cells with functional and repair benefits, including promoted cell survival, accelerated angiogenesis, and coordination of the inflammatory response after MI [6, 8, 30]. However, one of the primary barriers limiting the effectiveness of cell transplantation is the harsh ischemic microenvironment in which most of the injected cells would be killed by the inflammation and hypoxia. The discovery of cell-free components capable of developing analogous cell responses in target cells, such as exosomes [31]. Exosomes provide a promising alternative approach to overcome the disadvantages without a significant immune response. Once exosomes are secreted into the extracellular system, they are stable for a relatively long period of time and can transfer cell-specific signature signaling molecules to the target or recipient cells [32]. In this study, to overcome the limitations of cell therapy, we isolated the exosomes from GATA4-overexpressing cCFU-Fs in conditioned medium. We used exosome-specific markers, electron microscopy, and nano-detection techniques to confirm that the vesicles we used were exosomes. We utilized exosome-specific markers, electron microscopy, and NanoSight detection to confirm that the vesicles we use are exosomes. Interestingly, we found that compared to NC-Exo, the expression of Alix (ALG-2-interacting protein X) in GATA4-Exo was significantly reduced. Alix, also known as AIP1, is a cytoplasmic protein ubiquitously expressed and concentrated in phagosomes and exosomes [33]. Studies have shown that Alix can not only regulate the formation of vesicles inside MVB, but also regulate caspase-dependent and independent cell death, and this function is under the tight control of apoptosis-linked gene 2 (ALG-2), a Ca^2+^-binding protein [34]. Alix over-expression has been reported to enhance detachment-induced death of HeLa cells [35] and induce apoptosis in neurons [36]. Caspase activation by Alix followed by cell death was demonstrated upon over-expression of the protein in post-mitotic cerebellar granule neurons [37]. Coincidentally, the present study showed that the anti-apoptotic effect of GATA4-Exo weakly expressing Alix is more obvious. Hence, it is reasonable to hypothesize that Alix may be involved in the cytoprotective effects exert by GATA4-Exo, which deserves our further study.

GATA4 is a very important transcription factor in the early stage of heart development and regulates the differentiation, growth, and survival of a wide range of cell types [38, 39]. In addition, GATA4 has been shown to be important in endothelial cell maturation/differentiation, as shown mainly by the observation of defective tube formation and the appearance of CD31 low endothelial cells in Tie2 CreERT2 transgene-mediated GATA4-deleted mice [15]. Here, we used a genetic approach to generate cCFU-Fs constitutively overexpressing GATA4 and showed that these cells have dramatically increased cardiogenic potential compared to a GFP-expressing control cells. Considering that genetically modified cardiac-derived cells have been widely investigated in previous studies, it has been hypothesized that exosomes derived from re-edited cCFU-Fs can be used as ideal vehicles for gene delivery to facilitate gene and cell therapy. Here, we demonstrated that GATA4-Exo could confer better protective effects against hypoxia-induced myocardial cell injury. Our studies demonstrated that GATA4-Exo showed great potential in reducing CM apoptosis, reducing fibrosis area and improving cardiac function. Our results are also in agreement with earlier findings suggesting that GATA4-based gene transfer represents a novel and efficient therapeutic approach for the treatment of heart failure [40].

This work further expands our understanding of the underlying molecular basis for cardioprotection conferred by GATA4-Exo. Accumulating evidence suggests that exosomes serve as vectors for miRNA communication between different cell types [41]. Exosome-derived miRNAs can downregulate the expression of target genes in their recipient cells, which is a significant signaling transfer mechanism among neighboring cells [42]. We postulate that GATA4-Exo-specific miRNAs are involved in the inhibition of myocardial apoptosis. Indeed, miRNA profiling of cCFU-F^NC^- and cCFU-F^GATA4^-derived exosomes revealed that ten miRNAs were more highly expressed in GATA4-Exo compared with NC-Exo, including the antiapoptotic miRNA221. miR221 and its family members are highly expressed in various cancer-derived cells, including PC3 prostate carcinoma cells, HeLa cells, and thyroid carcinoma cells [43, 44]. Additionally, overexpression of miRNA221 was found to reduce ischemia/reperfusion (I/R)-induced apoptosis and autophagy in H9c2 cells and downregulate multiple apoptosis- and autophagy-related targets at the mRNA and protein levels in H9c2 cells [45]. Therefore, we focused on miR221 in this study to investigate the beneficial effect of GATA4-Exo-mediated cardioprotection. Here, our data demonstrate that both in vivo and in vitro miR221 mimics could protect cardiocytes against hypoxia-induced injury and the protective effect of GATA4-Exo was partially abolished by miR221 inhibitor. Moreover, miR221 was significantly increased in H9c2 cells cultured with GATA4-Exo compared with those cultured with NC-Exo which indicates that GATA4-Exo confer cardioprotection, at least in part, by miR221.

Among the predicted target genes, we focused on the tumor suppressor gene PTEN, which can antagonize PI3K activity by dephosphorylating PIP3 and thereby negatively regulate the activity of the Akt pathway [46, 47]. Activation of AKT is correlated with increased proliferation, metabolism, cellular migration, and apoptosis resistance [48, 49]. Recently, studies have shown that PTEN is also associated with apoptosis of myocardial cells after myocardial infarction [26, 50]. Ha et al. reported that the activation of PI3K/Akt signaling is involved in the improvement of cardiac function, reduction of infarct size, and decrease in myocardial apoptosis following myocardial I/R injury [51, 52]. A luciferase reporter assay confirmed that PTEN is a target gene of miR221. Modulation of miR221 expression by antisense oligonucleotides or overexpression strategies directly affected PTEN expression. Moreover, herein, we showed that the PTEN RNA expression level in GATA4-Exo was significantly lower than that in NC-Exo. Furthermore, we demonstrated that GATA4-Exo upregulated p-Akt but downregulated caspase-3, which is consistent with the findings of a previous study showing that increased PTEN expression in cultured neonatal rat primary CMs caused CM apoptosis mediated by increased caspase-3 [26].

## Conclusion

In this study, we used a genetic modification technique to promote the paracrine function of cCFU-Fs for repair of the ischemic myocardium. Exosomes derived from GATA4 overexpressed cCFU-Fs prevent cardiomyocyte apoptotic program, at least partly, via anti-apoptotic miRNAs contained therein. Our data provide compelling evidence of the role of GATA4-Exo in improving the healing response following injury. Therefore, exploring the active ingredients and functional mechanisms of GATA4-Exo is of great significance to developing a new biotherapy of myocardial ischemia based on exosomes. This study offered a new approach to alleviating myocardium ischemic injury.

## Supplementary information

**Additional file 1: Fig S1.** Real-time PCR was performed to quantify the expression of miR221 in left ventricular tissue after sham-, PBS-, GATA4-Exo- or NC-Exo-treated. (*n*=6, **p< 0.01, *p<0.05 for comparisons between groups.)

**Additional file 2: Fig S2.** The expression of GATA4 in NC-Exo and GATA-Exo detected by real-time PCR. *p<0.05 *n*=3

**Additional file 3: Fig S3.** miR221 is involved in the cardioprotection effect of GATA4-Exo after myocardial infarction. (a, b) Representative M-mode images and quantification of EF% and FS% measured by echocardiography of sham, PBS, GATA4-Exo,NC-Exo, miR221 mimics, and GATA4-Exo+miR221 inhibitor -treated mice at 28 days after MI. (*n*=6, **p< 0.01, *p<0.05 for comparisons between groups.) (c, d) Masson trichome-stained myocardial sections at 28 days after MI in mice treated with PBS, GATA4-Exo,NC-Exo, miR221 mimics, and GATA4-Exo+miR221 inhibitor. (n=6 mice per experimental group, for each sample, 4 to 6 slices were taken according to the size of the heart, **p< 0.01, *p<0.05 for comparisons between groups.) Scale bar: 1000μm. (e) Immunohistochemistry of sham, PBS, GATA4-Exo,NC-Exo, miR221 mimics, or GATA4-Exo+miR221 inhibitor -treated heart sections marking TUNEL-positive cardiomyocytes within the border zone of infarcted hearts at 24 h after LAD artery ligation. Scale bar: 50 μm. (f) Quantification of myocardial apoptosis. Green staining indicates TUNEL-positive cells (*p<0.05, **p< 0.01 between the indicated groups, n=6). (g,h) The protein expression levels of c-caspase-3 within the myocardium. Relative expression was determined following normalization to β-actin levels (*p<0.05, **p< 0.01 between indicated groups, n=6).

## Data Availability

miRNA array analysis data for GATA4-Exo and NC-Exo have been deposited in the NCBI Gene Expression Omnibus (GEO) database according to the MIAME guidelines (accession number GSE141092). The other datasets generated and/or analyzed during the study are available from the corresponding author on reasonable request.

## Abbreviations

cCFU-Fs: Cardiac colony-forming unit fibroblasts
GATA4: GATA-binding protein 4
MSC: Mesenchymal stem cell
PTEN: Phosphate and tension homology deleted on chromosome ten
LV: Left ventricular
MI: Myocardial infarction
LAD: Left anterior descending
