# Supplementary figures and images for: Overexpression of GATA4 enhances the antiapoptotic effect of exosomes secreted from cardiac colony-forming unit fibroblasts via miRNA221-mediated targeting of the PTEN/PI3K/AKT signaling pathway
Source: Stem Cell Res Ther. 2020 Jun 26;11:251. doi: 10.1186/s13287-020-01759-8 (PMC7318537; doi:10.1186/s13287-020-01759-8)

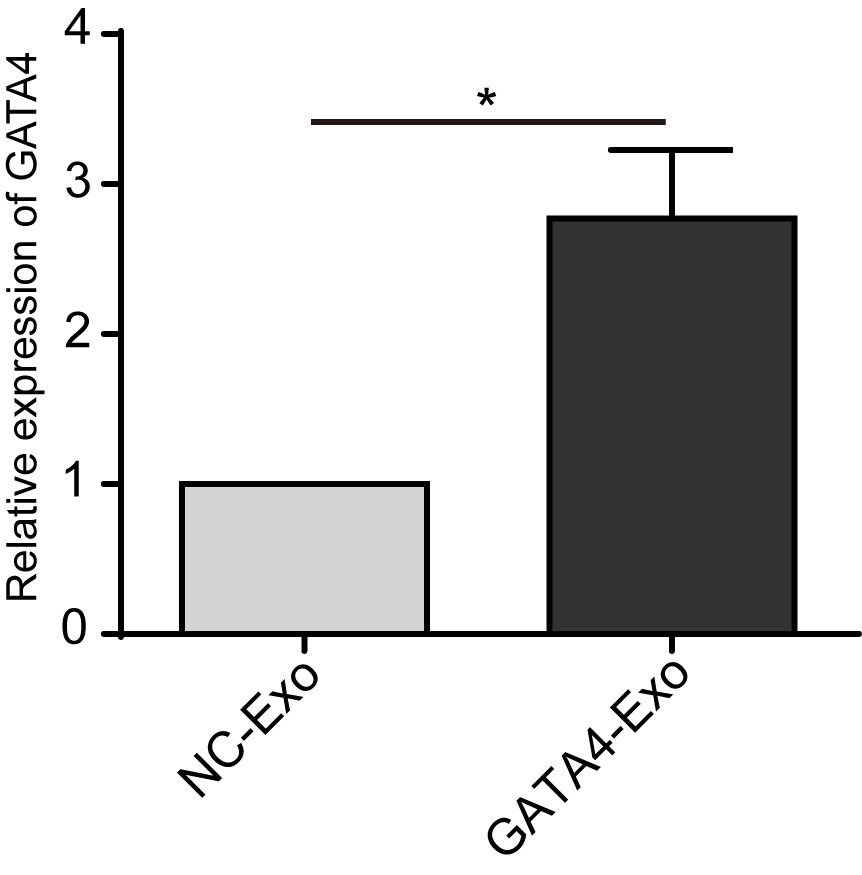

Supplement: Supplementary file 1 — Additional file 1: Fig S1. Real-time PCR was performed to quantify the expression of miR221 in left ventricular tissue after sham-, PBS-, GATA4-Exo- or NC-Exo-treated. (n=6, **p< 0.01, *p<0.05 for comparisons between groups.) [file 13287_2020_1759_MOESM1_ESM.tif]

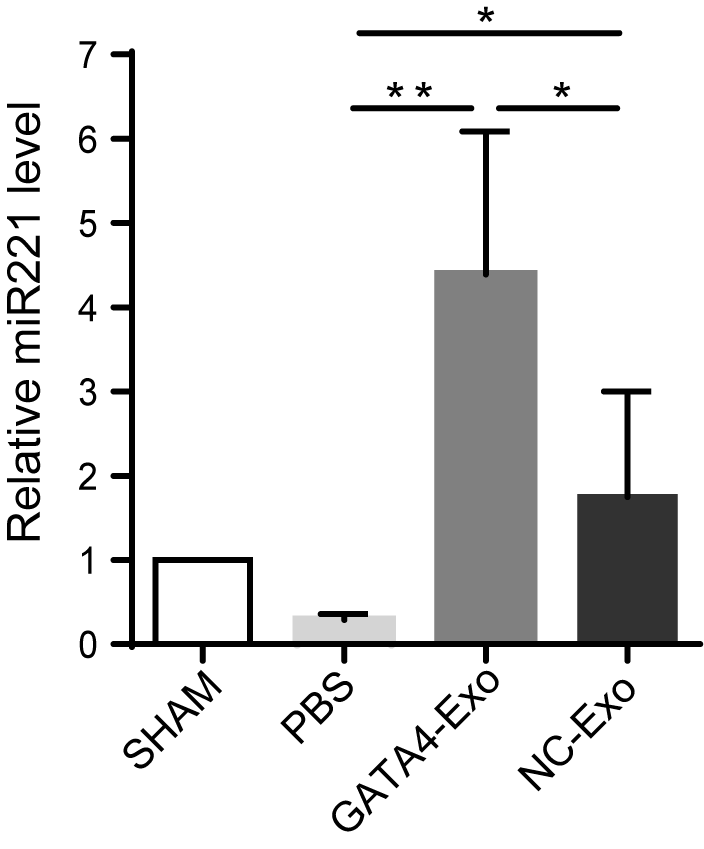

Supplement: Supplementary file 2 — Additional file 2: Fig S2. The expression of GATA4 in NC-Exo and GATA-Exo detected by real-time PCR. *p<0.05 n=3 [file 13287_2020_1759_MOESM2_ESM.tif]

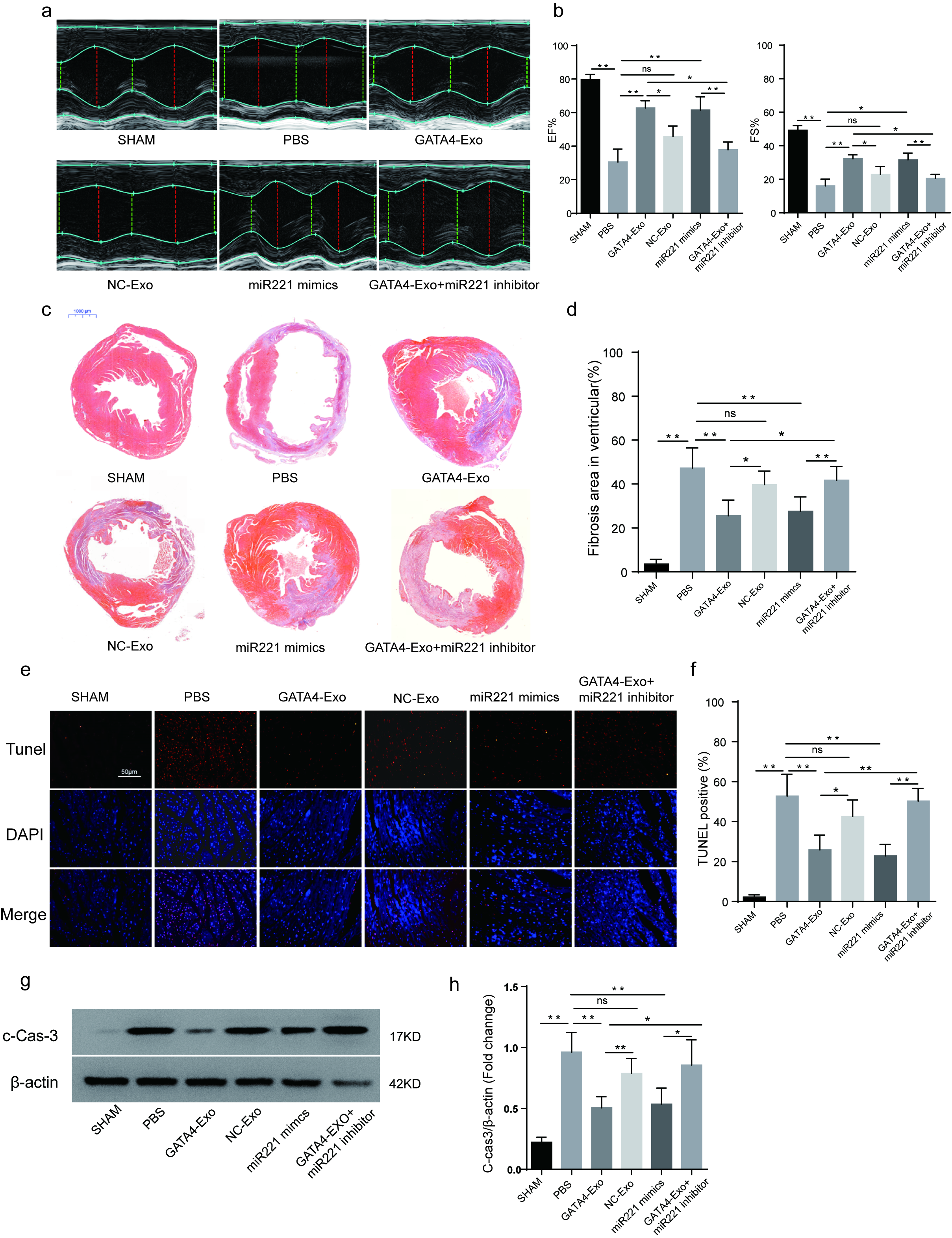

Supplement: Supplementary file 3 — Additional file 3: Fig S3. miR221 is involved in the cardioprotection effect of GATA4-Exo after myocardial infarction. (a, b) Representative M-mode images and quantification of EF% and FS% measured by echocardiography of sham, PBS, GATA4-Exo,NC-Exo, miR221 mimics, and GATA4-Exo+miR221 inhibitor -treated mice at 28 days after MI. (n=6, **p< 0.01, *p<0.05 for comparisons between groups.) (c, d) Masson trichome-stained myocardial sections at 28 days after MI in mice treated with PBS, GATA4-Exo,NC-Exo, miR221 mimics, and GATA4-Exo+miR221 inhibitor. (n=6 mice per experimental group, for each sample, 4 to 6 slices were taken according to the size of the heart, **p< 0.01, *p<0.05 for comparisons between groups.) Scale bar: 1000μm. (e) Immunohistochemistry of sham, PBS, GATA4-Exo,NC-Exo, miR221 mimics, or GATA4-Exo+miR221 inhibitor -treated heart sections marking TUNEL-positive cardiomyocytes within the border zone of infarcted hearts at 24 h after LAD artery ligation. Scale bar: 50 μm. (f) Quantification of myocardial apoptosis. Green staining indicates TUNEL-positive cells (*p<0.05, **p< 0.01 between the indicated groups, n=6). (g,h) The protein expression levels of c-caspase-3 within the myocardium. Relative expression was determined following normalization to β-actin levels (*p<0.05, **p< 0.01 between indicated groups, n=6). [file 13287_2020_1759_MOESM3_ESM.tif]
